# Response to Commentary: Autoimmune/Autoinflammatory Syndrome Induced by Adjuvants (ASIA Syndrome) After Polypropylene Mesh Implantation - Protocol of a Pilot Study for Diagnostics and Treatment

**DOI:** 10.3389/jaws.2025.15811

**Published:** 2026-01-12

**Authors:** W. A. R. Zwaans, M. J. C. A. M. Gielen, N. D. Bouvy, C. R. Kowalik, R. M. H. Roumen, M. C. Slot, J. P. W. R. Roovers

**Affiliations:** 1 SolviMáx Centre of Expertise for Complex Groin and Abdominal Wall Pathology, Eindhoven, Netherlands; 2 Department of Surgery, Máxima Medical Centre, Veldhoven, Netherlands; 3 NUTRIM School of Nutrition and Translational Research in Metabolism, Maastricht University, Maastricht, Netherlands; 4 Research Consortium Mesh, Utrecht, Netherlands; 5 GROW Research Institute for Oncology and Reproduction, Maastricht University, Maastricht, Netherlands; 6 Department of Surgery, Maastricht University Medical Centre, Maastricht, Netherlands; 7 Department of Surgery, Leiden University Medical Center, Leiden, Netherlands; 8 Department of Obstetrics and Gynaecology, Amsterdam University Medical Centres, Amsterdam, Netherlands; 9 Amsterdam Reproduction and Development Research Institute, Amsterdam, Netherlands; 10 Bergman Clinics, Amsterdam, Netherlands; 11 Department of Allergology and Clinical Immunology, Maastricht University Medical Centre, Maastricht, Netherlands

**Keywords:** ASIA syndrome, polypropylene, implant reaction, mesh complications, systemic autoimmune disorders

With great interest we have read the Commentary on the published protocol of our study named “Autoimmune/Autoinflammatory Syndrome Induced by Adjuvants (ASIA Syndrome) After Polypropylene Mesh Implantation - Protocol of a Pilot Study for Diagnostics and Treatment.” Several of the comments have led us to clarify more on the considerations that were taken into account during the study’s design, as well as extra details not mentioned in the previous manuscript. We anticipate this elaboration can resolve remaining ambiguities and any lasting uncertainties on the study’s methodological rigor.

Autoimmune Syndrome Induced by Adjuvants (ASIA) remains a debated and largely hypothetical entity, with current evidence insufficient to confirm its validity as a distinct clinical condition. Published reports supporting ASIA after polypropylene mesh implantation have primarily consisted of case series, which by design cannot establish causality between polypropylene mesh implants and the syndrome. Investigations of immunological parameters in patients with polypropylene mesh exposure have not demonstrated definitive autoimmune responses [1], and systematic reviews to date have also failed to establish an association between these implants and autoimmune disease [2, 3]. Nonetheless, ongoing concern expressed by patients and regulatory authorities underscores the need for continued research. A significant challenge lies in the provisional ASIA criteria, which are broad, lack temporal specificity, and risk encompassing patients with non-specific symptoms, such as fatigue, even decades after exposure.

As established in the literature, polypropylene mesh degradation *in vivo* is a documented phenomenon [4–7]. Consequently, surgeons with experience in performing revisional surgery for mesh-related complications can confirm that complete removal of all polypropylene microparticles is unattainable in clinical practice; aggressive attempts to do so may cause significant patient morbidity. Therefore, attributing persistent symptoms solely to these residual microscopic particles represents an oversimplification of a complex clinical picture. A more plausible causal relationship would be supported by the presence of a dose-response effect; specifically, a discernible improvement in symptoms following macroscopic mesh explantation would be expected. Regarding pain relief, clinical evidence indicates that partial mesh resection is as effective as complete resection, with a lower complication rate [8].

Besides degradation, mesh implants have been proven to be biologically active in earlier (animal) studies through a foreign body response, which is a well-known concept in immunology. Polypropylene is no exception to this concept. Presence of this implant, regardless of its quantity, can evoke a locoregional immune response initially, which leads to the desired effect of tissue integration and fibrosis to the mesh, and strengthening of the tissue in surgical repairs [9]. Although the acute response leads to most changes, this response is rapidly self-limiting and most long-term complications of mesh are a direct result of local friction and irritation of the implant’s surface or location. Therefore, the proposed dose-response effect on symptoms is not on immunologic grounds, but on mechanical grounds, in which surgery could help relieve symptoms by decreasing the mechanical irritation.

As noted in the commentary, we agree that sIL2R remains a relevant marker for analysis of immunological disorders. In the immunological blood panel that will be routinely conducted for all patients, sIL2R is included but was not mentioned in the previous manuscript. We consider angiotensin-converting enzyme (ACE) to be an irrelevant marker for autoimmune reactions outside of pulmonary sarcoidosis, based on its limited evidential support in the literature and its limited added value within the context of other immunologic tests [10, 11].

Despite the acknowledged limitations and ongoing controversy surrounding mesh allergy testing (MAT), our pilot study aims to assess its potential diagnostic utility within a specific patient cohort. In our evaluation, MAT results may inform clinical decisions regarding mesh removal, and in two cases, the test contributed to the decision to proceed with surgical mesh removal.

The primary outcome will be symptom relief following either explantation or follow-up. Consequently, the follow-up period was appropriately defined from the time of mesh removal (or study inclusion). This design directly assesses the intervention’s effect, making the delayed onset of ASIA symptoms, a feature of pathogenesis, not a relevant factor for measuring postoperative improvement.

In light of our current clarifications on the most important objections made by the authors, who publish high numbers of articles on ASIA-syndrome, we deem our response sufficient to resolve remaining qualms on the study’s methodological rigor and integrity. Finally, we hope our study’s results can clarify whether any immunologic basis, be it in extensive blood work or through MAT, can be found for patients suffering from complaints included in the ASIA diagnostic criteria, or whether the complaints should be attributed elsewhere than as an auto-immune reaction to the surgically implanted materials.
